# Gallbladder edema as a clue to zolbetuximab-associated protein-losing enteropathy in gastric cancer: a case report

**DOI:** 10.1007/s10120-025-01668-w

**Published:** 2025-09-30

**Authors:** Yoshihiko Kakiuchi, Shinji Kuroda, Shunya Hanzawa, Nobuhiko Kanaya, Hajime Kashima, Satoru Kikuchi, Kunitoshi Shigeyasu, Yoshiyasu Kono, Shunsuke Kagawa, Toshiyoshi Fujiwara

**Affiliations:** 1 Department of Gastroenterological Surgery, Dentistry and Pharmaceutical Sciences, Okayama University Graduate School of Medicine, Okayama, Japan; 2 Department of Gastroenterology and Hepatology, Dentistry and Pharmaceutical Sciences, Okayama University Graduate School of Medicine, Okayama, Japan

**Keywords:** Gastric cancer, Zolbetuximab, CLDN 18.2, Protein-losing enteropathy, Gallbladder edema

## Abstract

We report a rare case of protein-losing enteropathy (PLE) during zolbetuximab treatment in a 73-year-old woman with Stage IVB gastric cancer. After chemo-immunotherapy and curative surgery, 3rd-line treatment with capecitabine, oxaliplatin, and zolbetuximab was initiated due to recurrence. The patient developed persistent right upper abdominal pain; imaging revealed gallbladder wall edema, followed by mild gastric wall edema, despite unremarkable laboratory findings. Protein-losing scintigraphy demonstrated abnormal gastric protein leakage, leading to a diagnosis of PLE. While gastrointestinal toxicity is known with zolbetuximab, this is, to our knowledge, the first clinically diagnosed case of PLE in which gallbladder edema served as a diagnostic clue. As treatment strategies for advanced gastric cancer grow increasingly complex, achieving maximum therapeutic benefit requires not only optimal drug selection but also timely recognition and management of adverse events. With the broader use of zolbetuximab, clinicians should be mindful of this rare but potentially significant complication.

## Introduction

Gastric cancer with distant metastases, including para-aortic lymph node (PAN) involvement, is classified as stage IVB and is typically treated with systemic chemotherapy. Although this stage is generally considered unresectable, the concept of oligo-metastatic disease has emerged as a clinically meaningful subset in which surgical intervention may be considered following a favorable response to neoadjuvant chemotherapy (NAC). The 7th edition of the Japanese Gastric Cancer Treatment Guidelines distinguishes NAC followed by surgery for oligo-metastasis cases from conversion surgery for initially unresectable cases, and weakly recommends the former. With the advent of novel agents, such as immune checkpoint inhibitors, including nivolumab and pembrolizumab, and zolbetuximab, an antibody targeting claudin-18 isoform 2 (CLDN 18.2), surgery may become a more viable option for select patients with oligo-metastasis. These agents also hold promise for improving outcomes in the setting of postoperative recurrence though their use requires careful management of potential adverse events (AEs). Here, we present a case of protein-losing enteropathy (PLE) associated with zolbetuximab, which was diagnosed during multimodal therapy after incidental detection of gallbladder edema.

## Case presentation

A 73-year-old woman was referred to our hospital after upper gastrointestinal endoscopy for persistent epigastric discomfort led to a diagnosis of gastric cancer. Further evaluation led to a diagnosis of unresectable advanced gastric cancer with metastases to the #16a1 lateral PAN in May 2023 (cT4aN3aM1 [LYM] Stage IVB). Due to signs of impending gastric outlet obstruction, she underwent diagnostic laparoscopy and laparoscopic gastrojejunostomy in a single operation. Systemic chemotherapy was initiated with S-1, oxaliplatin, and nivolumab. After five cycles, restaging computed tomography (CT) demonstrated complete resolution of the PAN metastases, and curative surgery was planned. Although the patient’s clinical condition remained stable, the same CT scan revealed interstitial pneumonitis consistent with a Grade 2 immune-related AE (irAE). Corticosteroid therapy was initiated, and as radiologic improvement was observed on chest X-ray and CT, the steroid dose was gradually tapered. Approximately two months later, a robot-assisted distal gastrectomy was successfully performed. The bypass was preserved, and D2 lymphadenectomy, including dissection of the #16a2 lateral node station, was completed. Pathological diagnosis revealed no residual primary tumor, with one metastatic lymph node out of three in station #4d, resulting in a final pathological stage of ypT0N1M0. Based on shared decision-making, postoperative chemotherapy was not administered, and the patient was placed under surveillance.

**Fig. 1:**
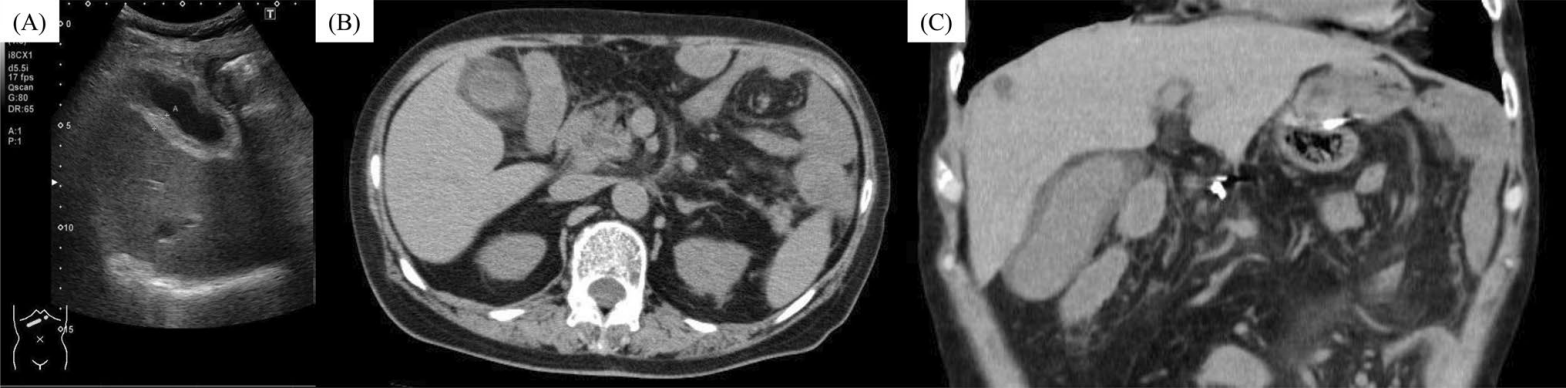
Imaging findings at the primary care physician at the onset of right upper abdominal pain. **A** Abdominal US showing gallbladder wall thickening of approximately 9 mm. **B**, **C** CT demonstrating diffuse thickening of the gallbladder wall without increased attenuation of the surrounding fat tissue

**Fig. 2:**
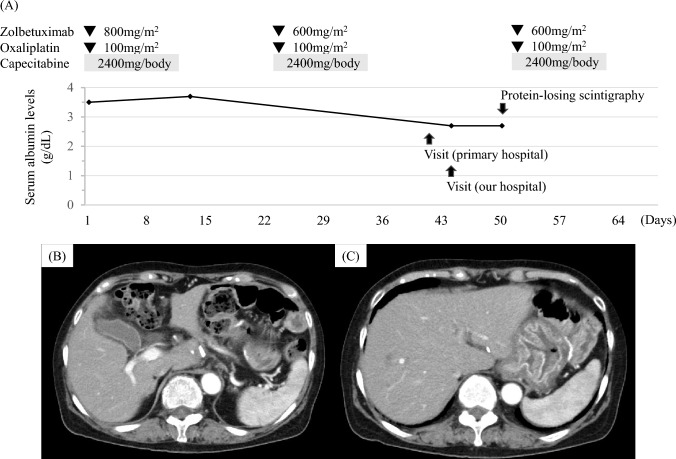
Clinical course and imaging findings at our hospital visit. **A** Timeline of drug administration, serum albumin levels, and diagnostic evaluations. Blood tests during the second cycle were performed at the primary care physician, where serum albumin was not measured. **B**, **C** Contrast-enhanced CT images at our hospital showing improvement of gallbladder wall edema, while new gastric wall edema became apparent

**Fig. 3:**
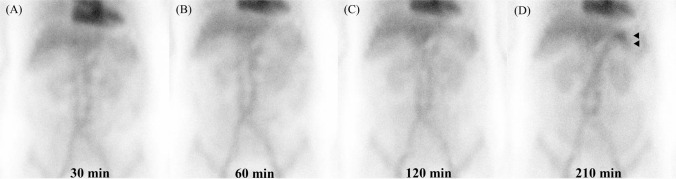
Findings of protein-losing scintigraphy. **A**–**C** Scintigraphic images obtained 30–120 min following intravenous injection of Tc-labeled tracer. **D** Abnormal tracer leakage from the stomach is noted at 210 min (arrow)

In October 2024, eight months postoperatively, follow-up CT revealed local lymph node recurrence. Second-line chemotherapy with nab-paclitaxel and ramucirumab was initiated in response to disease recurrence, as continuation of the 1st-line regimen, despite its effectiveness, was deemed inappropriate due to the development of an irAE. However, after four cycles, rising tumor markers and radiological evidence of progression led to a diagnosis of progression disease. Third-line treatment with capecitabine, oxaliplatin, and zolbetuximab was then initiated in March 2025. The 1st cycle was well tolerated. However, on day 19 of the 2nd cycle, the patient developed right upper abdominal pain and visited her primary care physician. Laboratory tests showed normal inflammatory markers and liver function, and abdominal ultrasound (Fig. 1A) and CT (Fig. 1B and C) showed gallbladder wall edema, but no findings supportive of consistent with cholecystitis. Based on these results, symptomatic management with analgesic was initiated. However, as the pain persisted and gradually spread across the entire upper abdomen, she visited our hospital on day 22. Blood tests revealed no significant change compared to those performed by her primary care physician; however, her serum albumin level, which had been 3.7 g/dL near the end of 1st cycle, had decreased to 2.7 g/dL (Fig. 2A). CT demonstrated improvement in gallbladder edema (Fig. 2B) but newly observed mild gastric wall edema (Fig. 2C). On day 28, protein-losing scintigraphy showed no abnormal findings from 30 to 120 min after tracer injection (Fig. 3A–C); however, protein leakage from the stomach was observed at 210 min (Fig. 3D). Based on these findings, a diagnosis of PLE was made. As the condition was not considered a treatment-intolerable AE, the 3rd cycle of chemotherapy was initiated the day after the scintigraphy (Fig. 2A). The patient’s pain gradually subsided, and treatment is currently ongoing.

## Discussion

According to the REGATTA trial [1], continued chemotherapy is the standard approach for patients with unresectable advanced gastric cancer. The ToGA trial [2] established trastuzumab as a 1st-line treatment for HER2-positive gastric cancer. Subsequently, the ATTRACTION-2 trial [3] led to the approval of nivolumab as a 3rd-line therapy. More recently, the CheckMate 649 [4] and ATTRACTION-4 [5] trials have expanded the role of nivolumab to a 1st-line treatment for HER2-negative gastric cancer. Despite these advancements, the range of 1st-line options has remained relatively limited. The SPOTLIGHT [6] and GLOW [7] trials have positioned zolbetuximab as a promising 1st-line agent for HER2-negative gastric cancer expressing CLDN 18.2. Similarly, the KEYNOTE-811 [8] trial has demonstrated the efficacy of combining trastuzumab and pembrolizumab as the 1st-line treatment for HER2-positive gastric cancer, marking a notable advancement. These developments mark significant progress but also add complexity to treatment selection, and thereby increasing the reliance on companion diagnostics and biomarker-driven strategies. The potential for serious irAEs further underscores the need for expertise in managing these therapies. As treatment options continue to expand, identifying patients with oligo-metastasis who may benefit from surgery, and ensuring appropriate management of recurrence, remains a clinical priority.

**Fig. 4:**
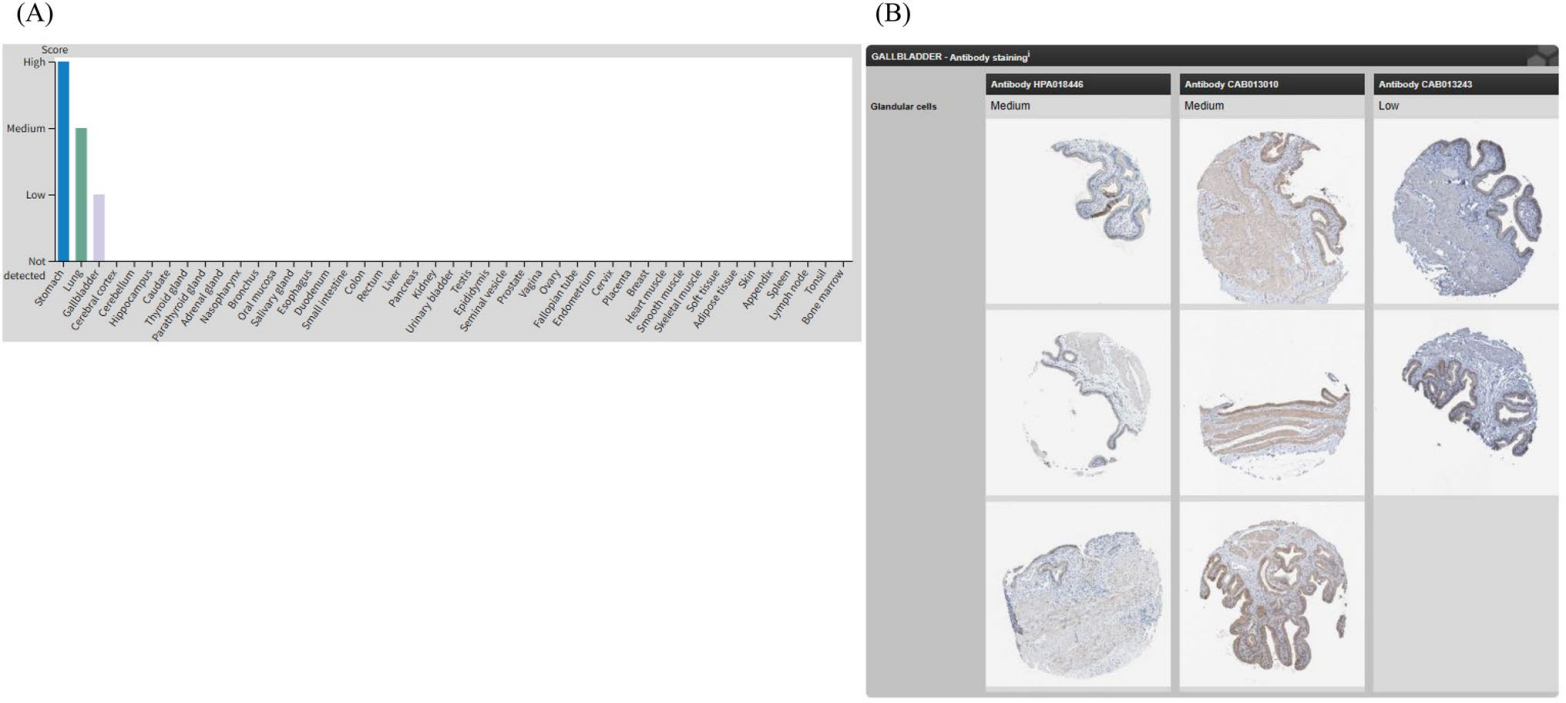
CLDN expression profile according to the Human Protein Atlas. **A** List of organs expressing CLDN. https://www.proteinatlas.org/ENSG00000066405-CLDN18/tissue. **B** Immunohistochemical staining of the gallbladder showing CLDN expression. https://www.proteinatlas.org/ENSG00000066405-CLDN18/tissue/gallbladder

We describe a case of PLE that emerged during zolbetuximab treatment, initially suspected due to incidental finding of gallbladder edema. Zolbetuximab is used as a 1st-line treatment for unresectable advanced or recurrent gastric cancer. Zolbetuximab targets CLDN 18.2, a tight junction protein critical for maintaining the gastric mucosal barrier [9, 10]. Its mechanism of action involves binding to CLDN18.2, thereby inducing antibody-dependent cellular cytotoxicity (ADCC) and complement-dependent cytotoxicity (CDC), which exert antitumor effects. Among the AEs, nausea and vomiting are well recognized; however, with its clinical use, a variety of additional AEs have been identified [6, 7]. These include hypoalbuminemia and gastritis. To date, reports of PLE during zolbetuximab therapy are extremely limited [11, 12]. Since CLDN 18.2 is also expressed in normal gastric mucosa, binding to these sites is thought to increase mucosal permeability, allowing the passage of protons and various other molecules [13]. This may lead to protein leakage resulting in hypoalbuminemia, while proton infiltration into the submucosal layer is considered to cause gastritis. Furthermore, according to the Human Protein Atlas portal (https://www.proteinatlas.org/), CLDN 18 protein expression is not limited to the stomach and lungs but is also observed in the gall bladder (Fig. 4A and B). To our knowledge, this is the first report to highlight gallbladder edema as a potential early diagnostic clue. Although scintigraphy did not show protein leakage from the gallbladder, the overall clinical course and findings strongly suggest an association between zolbetuximab and the observed PLE as the patient exhibited diffuse gallbladder wall edema rather than localized changes suggestive of peritoneal dissemination, and no findings indicative of cholecystitis were observed. The mechanism of gallbladder edema is often attributed to venous congestion associated with conditions such as hepatic failure or cardiac failure; however, no such signs were observed in this case. Instead, it is possible that decreased intravascular oncotic pressure secondary to hypoalbuminemia contributed to its development. According to the Human Protein Atlas, CLDN 18 expression in the gallbladder is observed at low levels. On the other hand, a previous report [14] has shown that the expression rate of CLDN 18 in gallbladder cancer exceeds 60% although it frequently exhibits heterogeneity. We considered that gallbladder edema may have preceded gastric edema. One possible explanation is that, within the context of intratumoral heterogeneity, certain regions might have shown relatively high expression levels. Notably, even when the gallbladder edema improved over time, gastric edema persisted, which may support the assumption that gallbladder edema developed earlier than gastric edema.

In the present case, curative surgery was performed following NAC for StageIV gastric cancer with oligo-metastatic PAN. Although the patient developed an irAE during the preoperative course, timely intervention enabled successful surgical resection. As surgical indications for oligo-metastatic gastric cancer are expected to increase, prompt and appropriate management of preoperative AEs, including irAEs, will be essential for optimizing outcomes. Moreover, patients may undergo adjuvant chemotherapy or systemic therapy upon recurrence, necessitating careful selection of agents and vigilant monitoring and management of associated toxicities. In this case, the patient developed gallbladder edema accompanied by right upper abdominal pain during treatment with zolbetuximab. Without accurate diagnosis, such a presentation could potentially be mistaken for cholecystitis, possibly leading to unnecessary interventions, including surgery. Thorough evaluation led to a diagnosis of PLE and allowed for the exclusion of cholecystitis, enabling continued administration of zolbetuximab. This highlights the importance of precise assessment in managing novel treatment-related toxicities to avoid premature discontinuation of effective therapies. PLE and the associated gallbladder edema observed in this case may emerge with increasing frequency as the use of zolbetuximab become more widespread.

In conclusion, as treatment strategies for advanced gastric cancer continue to grow more complex, achieving maximum therapeutic benefit requires not only appropriate drug selection but also timely recognition and management of AEs. This report underscores the importance of recognizing such potential toxicities and highlights the need to consider PLE and gallbladder edema as relevant AEs in the context of zolbetuximab therapy.
